# Evaluation of novel oral vaccine candidates and validation of a caprine model of Johne's disease

**DOI:** 10.3389/fcimb.2014.00026

**Published:** 2014-03-04

**Authors:** Murray E. Hines, Sue E. Turnquist, Marcia R. S. Ilha, Sreekumari Rajeev, Arthur L. Jones, Lisa Whittington, John P. Bannantine, Raúl G. Barletta, Yrjö T. Gröhn, Robab Katani, Adel M. Talaat, Lingling Li, Vivek Kapur

**Affiliations:** ^1^Tifton Veterinary Diagnostic and Investigational Laboratory, University of GeorgiaTifton, GA, USA; ^2^College of Veterinary Medicine, Food Animal Health and Management Program, University of GeorgiaAthens, GA, USA; ^3^National Animal Disease Center, United States Department of Agriculture, Agricultural Research ServiceAmes, IA, USA; ^4^School of Veterinary Medicine and Biomedical Sciences, University of NebraskaLincoln, NE, USA; ^5^Section of Epidemiology, Department of Population Medicine and Diagnostic Sciences, Cornell UniversityIthaca, NY, USA; ^6^Department of Veterinary Science, Penn State University, University ParkPennsylvania, PA, USA; ^7^Department of Pathobiological Sciences, University of Wisconsin-MadisonMadison, WI, USA

**Keywords:** *Mycobacterium avium* subsp *paratuberculosis*, vaccine efficacy, mutant vaccines, diagnostic tests, goats

## Abstract

Johne's disease (JD) caused by *Mycobacterium avium* subspecies *paratuberculosis* (MAP) is a major threat to the dairy industry and possibly some cases of Crohn's disease in humans. A MAP vaccine that reduced of clinical disease and/or reduced fecal shedding would aid in the control of JD. The objectives of this study were (1) to evaluate the efficacy of 5 attenuated strains of MAP as vaccine candidates compared to a commercial control vaccine using the protocol proposed by the Johne's Disease Integrated Program (JDIP) Animal Model Standardization Committee (AMSC), and (2) to validate the AMSC Johne's disease goat challenge model. Eighty goat kids were vaccinated orally twice at 8 and 10 weeks of age with an experimental vaccine or once subcutaneously at 8 weeks with Silirum® (Zoetis), or a sham control oral vaccine at 8 and 10 weeks. Kids were challenged orally with a total of approximately 1.44 × 10^9^ CFU divided in two consecutive daily doses using MAP ATCC-700535 (K10-like bovine isolate). All kids were necropsied at 13 months post-challenge. Results indicated that the AMSC goat challenge model is a highly efficient and valid model for JD challenge studies. None of the experimental or control vaccines evaluated prevented MAP infection or eliminated fecal shedding, although the 329 vaccine lowered the incidence of infection, fecal shedding, tissue colonization and reduced lesion scores, but less than the control vaccine. Based on our results the relative performance ranking of the experimental live-attenuated vaccines evaluated, the 329 vaccine was the best performer, followed by the 318 vaccine, then 316 vaccine, 315 vaccine and finally the 319 vaccine was the worst performer. The subcutaneously injected control vaccine outperformed the orally-delivered mutant vaccine candidates. Two vaccines (329 and 318) do reduce presence of JD gross and microscopic lesions, slow progression of disease, and one vaccine (329) reduced fecal shedding and tissue colonization.

## Introduction

There are conflicting data on the ability of current vaccines for Johne's disease (JD) to reduce shedding of *Mycobacterium avium* subspecies *paratuberculosis* (MAP), and killed “whole cell” MAP vaccines often generate serious local tissue reaction and cross-reactions to intradermal tests for *M. bovis.* This limits their usefulness for control programs (Rideout et al., 2003). To date, no vaccine has been developed that elicits an immune response that completely eliminates viable MAP from the host (sterile immunity). This is in part attributable to a lack of knowledge of the factors that regulate the immune response to MAP or to pathogenic mycobacteria in general (Flynn and Chan, 2001). Not all animals exposed to MAP progress to clinical disease, but it is unclear whether infection is never established in some animals or whether they develop an immune response that controls or eliminates the pathogen (Rideout et al., 2003). Experimental trials with vaccinated animals challenged with virulent MAP or by natural exposure have shown the beneficial effects of immunization (Saxegaard and Fodstad, 1985; Fridriksdottir et al., 2000; Reddacliff et al., 2006; Stringer et al., 2013). Vaccination has reduced the severity of lesions, reduced the frequency of clinical signs and delayed onset of disease (Harris and Barletta, 2001; Stringer et al., 2013). A vaccine that reduced the rate or eliminated infection and reduced or eliminated fecal shedding of the organism would be of tremendous value in JD control programs if it could reduce disease prevalence (Rideout et al., 2003). In addition to its use in a control program, an efficacious vaccine could significantly reduce production losses and premature culling or death losses that are associated with JD in dairy cattle (Benedictus et al., 1987; NAHMS, 1997; Lombard et al., 2005). Similar studies of losses associated with Johne's disease in beef, sheep and goat herds have not been reported, but losses in these production systems are also likely to be substantial.

Virtually all researchers using a JD challenge model have been using different species of animals, challenge parameters, doses of MAP, dosage intervals and strains of MAP. This dramatically increases the number of variables between studies and makes direct comparison of challenge and vaccine efficacy trials very difficult and often impossible. A standardized challenge model for use in vaccine efficacy trials is absolutely essential to be able to directly compare the efficacy of various vaccine formulations. A similar conclusion of the necessity of a standardized challenge model for vaccine efficacy studies was made in August 2005 at the International Colloquium for *Paratuberculosis* held in Copenhagen during the “Role of Vaccination” workshop session. The Johne's Disease Integrated Program (JDIP) Animal Model Standardization committee (AMSC) was created to address this issue and has published suggested international guidelines for JD challenge studies in multiple species including cattle, sheep, goats, deer and mice (Hines et al., 2007b).

Recent longitudinal JD studies presented at the 2010 JDIP annual meeting in Denver, CO have suggested that JD management and environmental control practices have reduced the incidence of JD, but are not likely to be successful in eradicating JD or reducing the incidence of JD to acceptable levels within dairy herds. Subsequently, most JD researchers are of the opinion that an effective vaccine which eliminates fecal shedding and prevents clinical disease is the best option for long term control of JD. This led to the development of the JDIP and USDA APHIS/NIFA sponsored JD vaccine project which consisted of three blinded phases. Phase I consisted of evaluating vaccine candidates within *in vitro* macrophage studies at two independent laboratories. Twenty-two JD vaccine candidates were submitted by JD researchers worldwide for enrollment in the Phase I project. Phase II consisted of evaluating the best JD vaccine candidates as determined from the Phase I project (8 vaccine candidates) within an *in vivo* mouse challenge model performed at two different laboratories. Phase III consisted of evaluating the best five performing JD experimental vaccines as determined by the results of Phase I and II within an *in vivo* goat MAP challenge model using the AMSC recommended parameters.

The objectives of the Phase III goat vaccine trial were first to evaluate the efficacy of five MAP attenuated vaccine strains against a commercially available MAP vaccine (Silirum®, Pfizer) using the protocols and endpoints proposed by the AMSC, and second to validate the AMSC Johne's disease goat challenge model (see Hines et al., 2007b). The design of this study allows for a direct “head to head” comparison of the efficacy of the new experimental vaccines included within the study to identify vaccine candidates for further development.

## Materials and methods

### Experimental design

Eighty goat kids were vaccinated orally twice at 8 and 10 weeks of age with one of the attenuated vaccine strains or once subcutaneously at 8 weeks with Silirum®, or an oral sham control vaccine consisting of pasteurized goat milk. Kids were challenged orally with a total of 200 mg pelleted wet weight MAP (approximately 2.0 × 10^9^ CFU) divided in 2 consecutive daily 100 mg doses (approximately 1.0 × 10^9^ CFU each) using bovine MAP isolate ATCC-700535 (K10-like MAP strain). All experimental and control groups had 10 kids/group. Table 1 lists the eight experimental and control groups with treatments. Blood and fecal samples were collected prior to vaccination (baseline samples) and similar samples were collected monthly throughout the study. Immunological tests evaluated include humoral response evaluation by AGID, ELISA, and cell mediated response by comparative purified protein derivative (PPD) skin testing (*M. avium*, Johnin, and *M. bovis* PPD's). Kids within each group were euthanized and necropsied at 13 months post-challenge. Gross and microscopic lesions and relative number of acid-fast bacilli were evaluated and scored at necropsy. Monthly fecal cultures and culture of selected necropsy tissues (tonsil, submandibular lymph nodes, liver, spleen, mesenteric lymph nodes, ileocecal lymph nodes, duodenum, jejunum, ileum, and spiral colon) were performed on Herrold's egg yolk medium (HEYM) and CFU/g determined for each specimen. Additional immunologic and diagnostic tests including AGID, ELISA, intradermal skin testing, fecal PCR and tissue PCR were performed throughout this study. Overall necropsy lesion score for each animal was calculated by adding the individual gross, microscopic and acid-fast stain scores as defined in Table 2.

**Table 1:** **Summary of experimental design and vaccines**.

**Experimental group**	**Vaccine (description/mutant insertion site)**	**Treatment**
Group 1 (10 kids)	Negative control (Sham vaccine)	Sham-vaccinated, unchallenged
Group 2 (10 kids)	Control vaccine (Silirum0® vaccine)	Vaccinated, challenged
Group 3 (10 kids)	316 vaccine (between MAP3695 and FadE5)^*^	Vaccinated, challenged
Group 4 (10 kids)	315 vaccine (MAP1566)^*^	Vaccinated, challenged
Group 5 (10 kids)	319 vaccine (MAP1566)^*^	Vaccinated, challenged
Group 6 (10 kids)	318 vaccine (between MAP0282c and MAP0283c)^*^	Vaccinated, challenged
Group 7 (10 kids)	329 vaccine (fabG2_2)^**^	Vaccinated, challenged
Group 8 (10 kids)	Positive control (Sham vaccine)	Sham-vaccinated, challenged

* Raúl G. Barletta mutant vaccine strains derived from K-10 by mutagenesis with IS1096 derived transposons.

** Adel M. Talaat mutant vaccine strain (Shin et al., 2006).

**Table 2:** **Scores for necropsy grading system categorized by lesion severity and presence of acid-fast bacilli (AFB)**.

**Severity**	**Gross**	**Microscopic**	**No. AFB**
None	0	0	0.00
Mild	4	1	0.25
Moderate	8	2	0.50
Severe	12	3	0.75

Individual severity scores for each of the three categories were summed to achieve the final lesion score for each kid at necropsy. Lowest and highest possible scores using this system are 0.0 and 15.75, respectively. The relative number of AFB is given the least weight in this system since both paucibacillary and pleuribacillary forms of JD are recognized in some affected animals without an apparent major difference in disease severity.

### Animals

The University of Georgia and Tifton Veterinary Diagnostic and Investigational Laboratory have AALAC accredited animal facilities. Animal care was performed in accordance with the procedures of the University of Georgia Institutional Animal Care and Use committee (UGA Animal Welfare Assurance # A3437-01). Two-month old kids were purchased from a farm with no previous history of Johne's disease where all the adult goats were test negative by MAP serum ELISA (Parachek®, Biocor Animal Health, Omaha, NE), AGID (New York State Animal Health Diagnostic Laboratory method—personal communication, Susan Stehman, Cornell University, NY) and fecal cultures were negative for MAP (this farm supplied the kids for the previous vaccine study—Hines et al., 2007a). The kids were moved to the animal facilities at the Tifton Veterinary Diagnostic and Investigational Lab, acclimated for 1 week and then randomly assigned to one of 8 groups of 10 kids. The “pen effect” was reduced by equalizing the distribution of kid sex and size between pens. Baseline fecal and blood specimens were collected and the initial PPD skin testing performed. The kids were vaccinated orally with the blinded attenuated JD vaccines from the Kapur^6^ laboratory using two doses 2 weeks apart as per instructions provided. Each provided vaccine stock solution was blended using a 1:4 ratio with pasteurized goat milk and each individual dose was drawn into a 5 ml syringe. Kids were allowed to nurse the contents from the syringe or the contents were gently expressed on the back of the tongue during administration. All kids were housed in a restricted biosafety animal facility (BSL-2). Kids within challenged groups were housed separately from non-challenged groups in similar BSL-2 treatment rooms and managed identically. No contact was allowed between challenged and non-challenged groups at any time during the study. Free choice coastal Bermuda hay was available to the kids throughout the study. All kids were supplemented daily with a 10% protein commercial goat ration with no added antibiotics at a mean rate of 450 gm/kid/day. Goats were dewormed at monthly intervals using a combination of Moxidectin (Fort Dodge Laboratories, Fort Dodge, IA, USA) and Albendazole (Valbasen, Pfizer Animal Health, New York, NY, USA) given orally. All kids were vaccinated upon receipt (2 months old) with commercially-available tetanus toxoid (Professional Biological Company, Denver, CO, USA) and multivalent clostridial vaccine (Ultrabac 7, Pfizer Animal Health, New York, NY, USA). Male kids were castrated prior to start of the study (2 months old). Monthly blood and fecal samples were collected from each kid as described in the protocol for each test performed. All kids were necropsied at approximately 13 months post-challenge.

### Organism

The MAP strain used in this study was a bovine isolate of MAP ATCC—700535 (K10-like MAP strain) recovered from experimentally infected calves at University of Pennsylvania (Ray Sweeney) to help insure that virulent organisms are used for challenge.

### Vaccine preparation and administration

The Silirum® vaccine was obtained directly from Pfizer Animal Health and administered as a single dose according to manufacturer's recommendations by subcutaneous injection. The five experimental vaccines were cultured to equivalent optical densities (0.50 OD) and blinded (Table 1) off-site at Penn State University before being shipped to UGA. After the goats in the project had acclimated for 5 days, the experimental vaccines were administered orally in two 5 ml doses 2 weeks apart in commercially pasteurized goat milk at approximately 1.0 × 10^8^ organisms per dose. The orally administered sham vaccines for the positive and negative control groups consisted of two 5 ml doses of commercially pasteurized goat milk.

### Challenge protocol

The challenge inoculum was grown in Middlebrook 7H9 liquid media containing OADC, supplemented with mycobactin J (Allied Monitor, Fayette, MO, USA) and 1% glycerol. All bacteria were freshly cultivated for approximately 12 weeks, and never refrigerated or frozen prior to use. Organisms were pelleted by centrifugation at 3000 × g for 10 min at RT in a pre-weighed large cone-bottomed centrifuge tube and the supernatant discarded. After draining the excess fluid, an accurate wet weight of the bacterial pellet was determined and the volume calculated to result in a final concentration of 20 mg organisms per ml (Hines et al., 2007a,b). The inoculum was vortexed at maximum output for 2–3 min with three glass beads to break up clumped bacilli. Whole commercially pasteurized goat's milk was added to 80% of final volume needed to make the inoculum palatable and mixed by inversion thoroughly immediately prior to use. All kids in all challenge groups were challenged at the same time period 3 weeks after the last dose of vaccine given. Each kid was allowed to nurse 5 ml of the inoculum (100 mg pelleted wet weight, approximately 1.0 × 10^9^ CFU) from a syringe or the inoculum was gently expressed on the back of the tongue. Similar doses of inoculum were prepared and given on the next day for a total of 2 doses (total of approximately 2.0 × 10^9^ CFU). The actual CFU of the inoculum was determined by dilution and plating on Middlebrook 7H10 agar plus OADC and mycobactin J, and the total of both doses was found to be 1.44 × 10^9^ CFU per challenged animal.

### Gross and microscopic lesions

A complete detailed necropsy was performed on each animal by board-certified veterinary pathologists. Tissue specimens obtained at necropsy were fixed in 10% buffered neutral formalin overnight, processed routinely and embedded in paraffin blocks. Serial sections were cut 4–6 μm thick and stained with H&E or Kinouyan's acid-fast stains. All specimens were described microscopically (blinded) by the same pathologist noting the presence, location and number of granulomatous lesions and presence and number of acid-fast bacilli. The combined gross and histologic descriptions for each animal were randomly scrambled, then blindly rated and given a lesion score by another veterinary pathologist unfamiliar with animal's group and the vaccines used within the study, but with extensive experience in the diagnosis of JD. The lesion rating system (Table 2; Hines et al., 2007a,b) was applied to the gross and histologic descriptions for each animal. Individual lesion severity scores for gross and microscopic lesions, and relative number of acid-fast bacilli, were summed to achieve the final lesion score for each kid at necropsy.

### Fecal and tissue culture

Monthly fecal specimens were cultured using the sedimentation method currently recommended by National Veterinary Services Laboratory (Whitlock et al., 2000) using Herrold's egg yolk medium (HEYM with ANV, BBL; Becton Dickinson and Co, Sparks, MD, USA) with and without supplementation with mycobactin J (Allied Monitor, Fayette, MO, USA). CFU obtained from 3 HEYM tubes were averaged and adjusted to represent final CFU per gram of feces.

Specimens of tonsil, submandibular lymph nodes, mesenteric lymph nodes, ileocecal lymph nodes, liver, spleen, duodenum, jejunum, ileum and spiral colon were obtained at necropsy for mycobacterial culture. Each tissue specimen was trimmed and weighed to achieve a 2 g specimen, decontaminated, blended in a stomacher and cultured as previously described (Hines et al., 1998, 2007a). A 0.5 ml aliquot of this tissue suspension was saved for tissue PCR analysis. HEYM tubes with and without mycobactin J were incubated at 37°C and observed weekly for a minimum of 16 weeks at which time visible colonies were counted. All positive mycobacterial cultures were confirmed by PCR targeting IS900 (Rajeev et al., 2005). CFU obtained from 3 HEYM tubes were averaged and adjusted to represent final CFU per gram of tissue (CFU/g).

### Fecal and tissue PCR

The DNA from monthly fecal specimens and tissues obtained from necropsy (tonsil, submandibular lymph nodes, mesenteric lymph nodes, ileocecal lymph nodes, liver, spleen, duodenum, jejunum, ileum, and spiral colon) were extracted using a Qiagen tissue DNA extraction protocol per manufacturer's instructions. PCR to detect MAP in feces and necropsy tissues targeting IS MAP02 (Stabel and Bannantine, 2005) was performed using the 4405545 AgPath-ID™ MAP (Johne's) Reagent Kit (Life Technologies/Albion, Grand Island, NY) with an ABI 7900 Real-Time Thermocycler in duplicate according to manufacturer's recommendations. Both cycle threshold (CT) values and positive/negative results were obtained on monthly fecal specimens and tissues collected at necropsy. CT values ≤37 were considered a positive result and inconclusive samples were repeated.

### PCR verification of MAP strains recovered from feces to evaluate persistence

To determine the persistence of the vaccine strains, primers were designed to amplify target regions specific to the knockout mutant yet able to distinguish vaccine from challenge strains based on product size. Colonies obtained from fecal culture slants of HEYM were picked and placed in 30 ul of distilled water and boiled for 5 min. The lysate was briefly centrifuged (13,000 × g, 1 min) and 5 ul was removed and used as template for amplification. Forward and reverse primers (Table 3) were added at 25 pmol each along with EconoTaq PLUS GREEN 2X master mix (Lucigen Corp). IS900 amplification was used as a positive control for all templates. The amplification conditions varied depending on the vaccine strain tested and were 94°C for 1 min followed by 25 cycles at 94°C for 30 s, 62°C for 30 s and 72°C for 2 min followed by a hold at 72°C for 5 min.

**Table 3:** **PCR primers used to detect survival of MAP mutant vaccine strains**.

**Mutant Strains**	**JDIP ID#**	**Name**	**Sequence (5′-3′)**	**Length**	**ORF**	**ORF**	**POI**	**WT amplicon**	**Mutant amplicon**
315/STM68	jdip005-F	MAP1566 Forward	GCTCTAGAGCTGGCATCAGGGCACTCAAGAAA	32	MAP1566	1074	1032	1179	3700 estimated
	jdip005-R	MAP1566 Reverse	CCCAAGCTTGGGTATTCGCTGCACAGCATGTCAGGT	36					
316/2E11	jdip006-F	2E11-IP-F	GCTGCAGCAACCAGCCGA	18	FadE5^1^	1836	137 bp upstream	1845	5241
	jdip006-R	2E11-IP-R	CCACCGTCACCGCAGGTAGA	20	MAP3695^1^	1074	30 bp upstream		
319/30H9	jdip009-F	MAP1566 Forward	GCTCTAGAGCTGGCATCAGGGCACTCAAGAAA	32	MAP1566	1074	272	1179	4575
	jdip009-R	MAP1566 Reverse	CCCAAGCTTGGGTATTCGCTGCACAGCATGTCAGGT	36					
318/40A940A9		AMT152	TTGCTCTTCCGCTTCTTCT	19	MAP0282c	792	N/A	N/A	N/A
			(sequencing primer)		MAP0283c^2^	513			
329/fabG2_2^*^	jdip019_R1	AMT 38	GTA ATA CGA CTC ACT ATA GGG CNN NNC ATG						
	jdip019_R1	AMT 858	TGC AGC AAC GCC AGG TCC ACA CT						
	jdip019_R2	AMT 39	TAA TAC GAC TCA CTA TAG GG						
	jdip019_R2	AMT 859	CTC TTG CTC TTC CGC TTC TTC TCC						

1 These genes are encoded in opposite directions, away from the POI.

2 Transposon lost upon further replication.

* Shin et al. (2006).

### Serologic tests for MAP

Serum collected from the goats pre- and post-vaccination and inoculation, and monthly thereafter, was evaluated by AGID and ELISA. Reagents and methods of the Johne's AGID protocol at the New York Animal Health Diagnostic Laboratory (NYAHDL) were used for AGID testing (personal communication, Susan Stehman, Cornell University, NY, USA). ELISA testing was done using a commercially-available USDA approved ELISA kit for goats (Parachek®, Biocor Animal Health, Omaha, NE, USA), according to manufacturer's recommendations.

### Intradermal skin testing

Standard *M. bovis* PPD (serial # 10033X), *M. avium* PPD (serial # 30-EXP-0303) and Johnin PPD (Johnin OT serial # 133–8705) were obtained from NVSL (Ames, IA, USA). One-tenth ml of each PPD was injected intradermally using standard tuberculin syringes on previously clipped skin of the cervical region. Injection sites were marked with a black marker pen for easier determination of injection site location. The kids were intradermal tested prior to vaccination, just prior to challenge, and again prior to necropsy. The cervical side was alternated between each series of PPD skin tests. The response to the PPD injections (skin thickness/induration) was measured using calipers at 72 h post-injection.

### Statistical analysis

Univariate analysis at each time point was conducted, to see if there were any differences between groups in the outcome measure. Because the data were sparse in some months, Fisher's Exact Test in PROC FREQ of SAS (SAS Institute, 2009) was used for categorical analyses (e.g., whether a goat tested Positive or Negative in a particular month). PROC UNIVARIATE of SAS (SAS Institute, 2009) was used to determine the mean and median values of numerical variables (e.g., culture CFU) in a particular month.

However, because the time points are not independent from each other, a mixed model, accounting for the correlation between months, was used [PROC MIXED in SAS (SAS Institute, 2009)] for numerical outcomes. Two sets of models were fitted, the first including all months, and the second excluding months for which the values were all 0. Month was the repeated factor, Goat ID was the subject factor, and an autoregressive covariance structure (this assumes that measurements taken closer together in time are more highly correlated than those taken further apart in time) was used. For weight and BCS (only one outcome), general linear models (PROC GLM of SAS, SAS Institute, 2009) were used. PROC GLM was also used to look at, e.g., CFU within each month. Pairwise differences between groups were studied.

PROC LIFETEST of SAS (SAS Institute, 2009) was used to generate Kaplan-Meier plots, with group as the stratification variable. Time (months) to the first Positive result was of interest. If the goat had all negative results (i.e., still tested negative at Month 15), it was censored (i.e., did not experience the event of interest). However, some groups (1 and 2) did not have any events, so “the likelihood ratio test for strata homogeneity is questionable.” Therefore, this was run again, omitting Groups 1 and 2. Tests of Equality over Strata [Log-Rank, Wilcoxon, −2 Log (Likelihood Ratio)] indicated whether there were differences among the groups, with respect to time to first positive result.

Because many of the raw data values (e.g., CFU) have great variation, the natural logarithm of these values was taken, to normalize the data, and the above analyses were re-run. To overcome the large variation in absolute values, which can cause problems in parametric analysis, a non-parametric approach was used, based on ranks of the groups. Within each outcome measure and time point, the groups were ranked, from 1 = best-performing (e.g., low CFU, or low proportion of positive responses) to 8 = worst-performing (e.g., high CFU, or high proportion of positive responses). Overall ranks were then obtained for each outcome measure, over time. The analysis was done in SAS's PROC NPAR1WAY (SAS Institute, 2009). In all analyses, statistical significance was assumed at *P* < 0.05.

## Results

### Morbidity and mortality not attributable to johne's disease

All 80 kids completed the study. Only occasional mild injuries occurred during the 13 months in our BSL-2 facility. Infestation with anthelmintic resistant *Haemonchus spp.* nematodes initially necessitated a monthly deworming schedule using a combination of Moxidectin and Albendazole orally, but after 6 months intestinal nematodes were eliminated. Sporadic mild clinical cases of intestinal coccidiosis required periodic 10 day treatments of the entire flock (all groups, challenged or non-challenged) with Amprolium (Corid®, Merial Limited, Iselin, NJ, USA) administered in drinking water tanks. A few kids within all groups had low numbers of *Monezia spp.* tapeworms in the small intestine.

### Clinical signs attributable to johne's disease

No appreciable differences were detected between any of the treatment groups throughout the study in average daily weight gain evaluated just before necropsy (data not shown). Body condition score of goats in group 1 (negative control) was higher than that in groups 3 (316 vaccinated; *P* < 0.05) and 4 (315 vaccinated; *P* < 0.05). Body condition score was higher in goats in group 7 (329 vaccinated) than in goats in groups 3 (316 vaccinated), 4 (315 vaccinated), 5 (319 vaccinated), 6 (318 vaccinated), and 8 (positive control) (*P*-values all less than 0.05) (data not shown). Distinct clinical signs attributable to JD were not observed in any challenged kids during the first 11 months post-challenge. However, in the last few weeks of the study, one kid in group 4 given the 315 vaccine and 2 kids in group 6 given the 318 vaccine began to manifest mild clinical signs including weight loss, decreased body condition scores, rough hair coat and minimal diarrhea (pasty non-pelleted feces) and had body condition scores ≤2 consistent with JD. None of the challenged animals in the positive control group (8) or Silirum® vaccinated group (2) manifested obvious clinical signs of JD.

### Effects of vaccine on antemortem vaccination site reaction

Tissue reaction at the Silirum® vaccination site in group 2 kids was measured pre-vaccination, at 2 months, at 6 months and again at 13 months post-vaccination (data not shown). Reactions consisted of localized swelling of the subcutis and overlying skin with formation of a variable-sized firm subcutaneous nodule approximately 1–2 weeks post-vaccination that decreased in size over time, but persisted in most cases to the end of the study. Hair loss or ulceration at the injection sites were not detected. The one dimensional thickness of any subcutaneous nodule (vaccine granuloma) and overlying skin at the vaccination sites in the brisket region was measured using calipers. If a reaction was not detectable at a vaccination site by palpation, the average skin thickness of the injection site was used. Only a single Silirum® vaccinated kid (#30) developed abscessation with drainage at the injection site. The maximum size of the tissue reaction ranged between 25–30 mm and generally occurred at 1–2 weeks post-vaccination and regressed slowly over time. Vaccine tissue reactions of 7–10 mm were still present in 40% of Silirum® vaccinated kids at necropsy. Histopathology of these remaining vaccination reactions revealed a nodular granuloma comprised of granulomatous inflammation admixed with lymphocytes and plasma cells with occasional foci of necrosis surrounded by granulation tissue and fibrosis. None of the experimental vaccines were given by injection.

### Effects of vaccine on gross lesions

Kids in challenged groups 3–8 had moderate to severe gross lesions consisting primarily of moderate to marked mesenteric and ileocecal lymph node enlargement with numerous variable-sized (1–10 mm) cortical tan foci of granulomatous inflammation. Occasional kids in these groups had mild to moderate thickening of the ileal and jejunal mucosa, but obvious corrugation was not detected. Some kids in groups 3–8 had prominent gross enlargement of lymphatics with associated lymphangitis. Challenged kids in group 2 (Silirum® vaccinated) showed minimal to mild gross lesions consisting primarily of mild mesenteric and ileocecal lymph node enlargement with occasional small cortical 1–3 mm tan foci of granulomatous inflammation. Obvious thickening of the small intestinal mucosa in these groups was generally not evident.

### Effects of vaccine on tissue microscopic lesions

#### Tonsil and submandibular lymph nodes

Microscopic lesions consisting of variable but usually low numbers of microgranulomas (granulomas with less than approximately 30 macrophages) were detected in submandibular lymph nodes, as well as tonsil in occasional kids of the challenged groups. Only rarely were microgranulomas detected in submandibular lymph nodes or tonsil in kids given the Silirum® vaccine (group 2). No granulomatous lesions were detected in these tissues from kids within the non-challenged negative control group (group 1).

#### Mesenteric and ileocecal lymph nodes

The mesenteric and ileocecal lymph nodes of non-challenged control kids (group 1) often had mild to moderate lymphoid hyperplasia with mild medullary histiocytosis and scattered medullary clusters of eosinophils. Lesions in mesenteric and ileocecal lymph nodes of challenged kids varied in degree of severity but were generally similar. These lesions consisted of variable numbers of microgranulomas, scattered patchy foci of granulomatous inflammation containing occasional multinucleate Langhan's giant cells and variable numbers of acid-fast bacilli primarily within the cortex of the nodes. In more severe cases, the microgranulomas and patchy foci of granulomatous inflammation tended to coalesce to form large granulomas or sheets of granulomatous inflammation containing variable numbers of Langhan's multinucleate giant cells and more numerous acid-fast bacilli. The number of acid-fast bacilli was generally lower in lymph nodes from group 2 (Silirum® vaccinated) than in group 4 (315 vaccinated) (*P* < 0.05); and lower in groups 2 (Silirum®; *P* = 0.05), 3 (316 vaccinated; *P* < 0.05), and 7 (329 vaccinated; *P* < 0.05) than in group 8 (positive control).

#### Intestines

**Figure 1:**
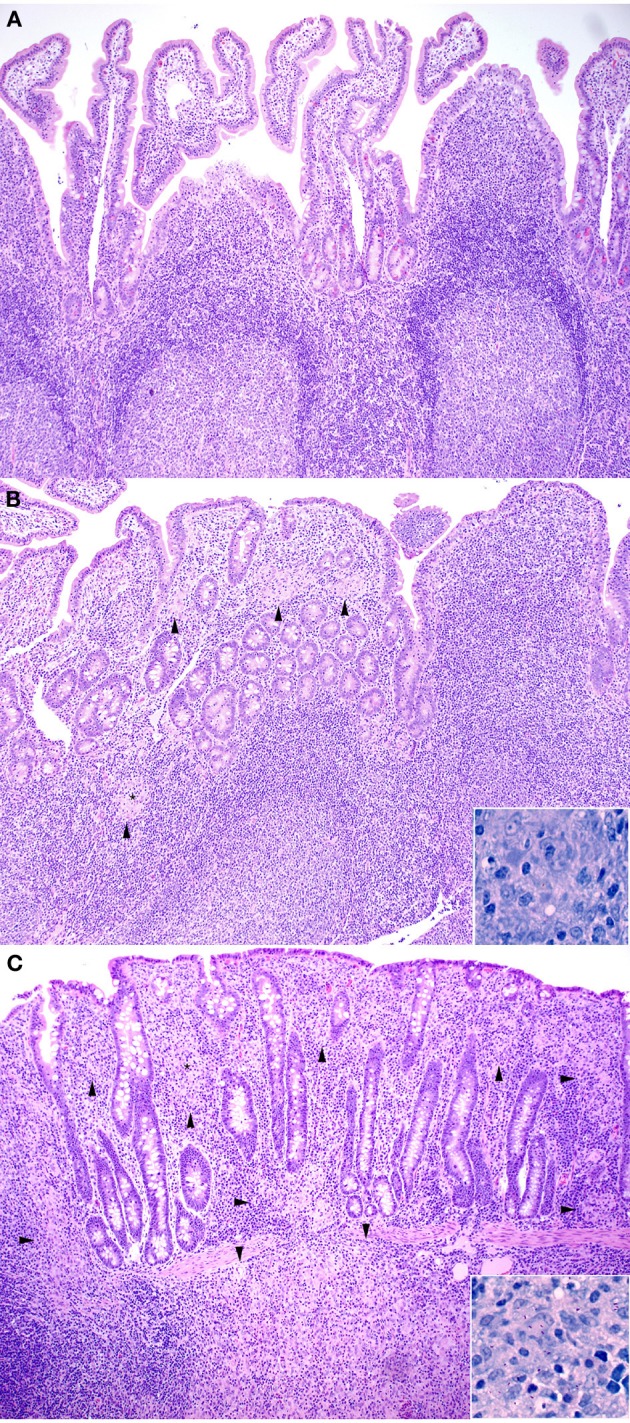
**(A–C)** Note how the severity of microscopic lesions increases along with the lesion score. Arrowheads indicate foci of granulomatous inflammatory infiltrate. **(A)** Representative Hematoxylin and Eosin stained section of non-challenged Sham vaccinated control ileum sample (100X). Lesion score = 0. **(B)** Hematoxylin and Eosin stained section of Silirum® vaccinated and challenged ileum sample (100X). Inset shows Kinouyan's acid-fast stain with a single acid-fast bacillus in center from area labeled with a small star (600X). Lesion score = 3.54. **(C)** Hematoxylin and Eosin stained section of higher lesion score in a 319 vaccine (group 5) ileum sample (100X). Inset shows Kinouyan's acid-fast stain with multiple acid-fast bacilli from area labeled by a small star (600X). Lesion score = 10.42.

**Figure 2:**
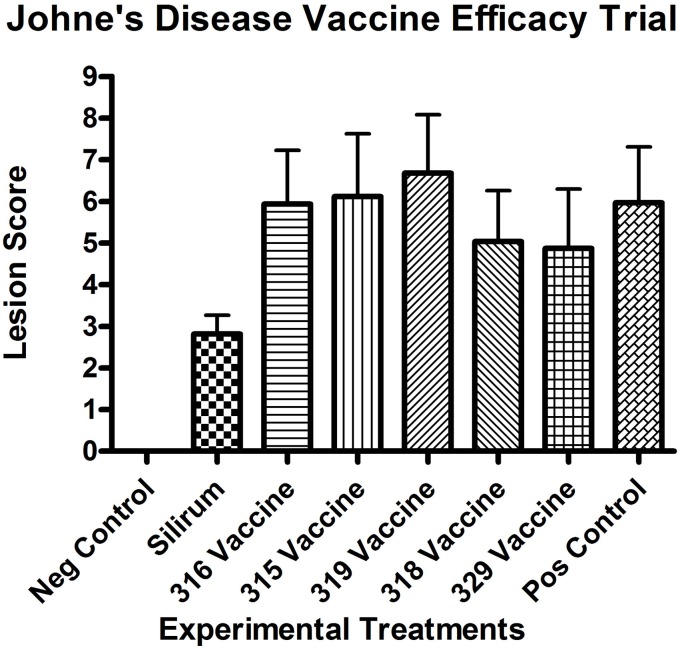
**Necropsy lesion scores for the challenged control and experimental groups at 13 months post-challenge.** All negative controls (group 1, non-challenged kids) had lesion scores of 0. (Error bars represent standard error of the mean).

All kids in all groups (challenged and non-challenged) had varying degrees (mild to moderate) of lymphoplasmacytic, eosinophilic and globule leukocyte infiltrate within all sections of small intestine consistent with parasitic enteritis (Figure 1A). No non-challenged kids in any group had granulomatous lesions or acid-fast bacilli compatible with JD. The small intestinal lesions attributable to JD consisted of microgranulomas, granulomas and patchy foci of granulomatous inflammation with epithelioid macrophages, variable numbers of multinucleate Langhan's giant cells and acid-fast bacilli within the mucosa, submucosa and Peyer's patches. The intestinal lesions were most severe in the terminal jejunum, less severe in the proximal jejunum and ileum and only evident in the duodenum within occasional more severely affected kids in groups 4 (315 vaccine), 5 (319 vaccine), and 8 (positive control). Lesion scores (Figure 2) were lower in group 2 (Silirum® vaccine) than in group 5 (319 vaccine; *P* < 0.05). Lesion scores were lower in groups 2 (Silirum®; *P* < 0.05), 6 (318 vaccine; *P* ≤ 0.05), and 7 (329 vaccine; *P* ≤ 0.05) than in group 8 (positive control). Variable degrees of lymphatic dilation and associated granulomatous lymphangitis were present in these groups. Most kids in group 2 given the Silirum® vaccine had lower numbers of microgranulomas, foci of granulomatous inflammation, Langhan's giant cells and acid-fast bacilli (Figure 1B). In groups 4 (315 vaccine), 5 (319 vaccine), and 8 (positive control) these lesions were of greater severity and granulomatous foci often coalesced to form large sheets of epithelioid macrophages containing numerous acid-fast bacilli and less Langhan's giant cells within the mucosa, submucosa and Peyer's patches of the small intestine (Figure 1C) and occasional small foci of granulomatous inflammation (microgranulomas) were present in the large intestine. The 329 vaccine showed the least lesion score among all the attenuated vaccines, although it was not statistically significantly different from the other attenuated vaccines.

#### Other organs

The liver was the only other organ where lesions and/or acid-fast bacilli compatible with JD were detected. The liver lesions in challenged kids consisted of variable numbers of microgranulomas, rare Langhan's giant cells and rare acid-fast bacilli that were present in most kids from all challenged groups. Liver microgranulomas were more common in groups 3–8 and only rarely present in group 2 (Silirum® vaccine) kids.

### Effects of vaccine on lesion score

All kids in all challenged groups had at least subtle lesions compatible with JD suggesting none of the vaccines completely abrogated infection. Only the control vaccine appeared to show a reduction in mean lesion score at 13 months post-challenge (Figure 2), compared with the positive control (*P* < 0.05). The 319 vaccine showed a slightly higher (+11.9%; *P* = 0.17) lesion score than the sham-vaccinated challenged positive control group (group 8), and the 318 and 329 vaccine groups (groups 6 and 7) showed a slightly lower lesion score [−15.7% (*P* < 0.05) and −18.4% (*P* < 0.05) respectively] than group 8. Mean lesion scores from groups 3–8 did not appear statistically different from each other.

### Effects of vaccine on fecal shedding of MAP

**Figure 3:**
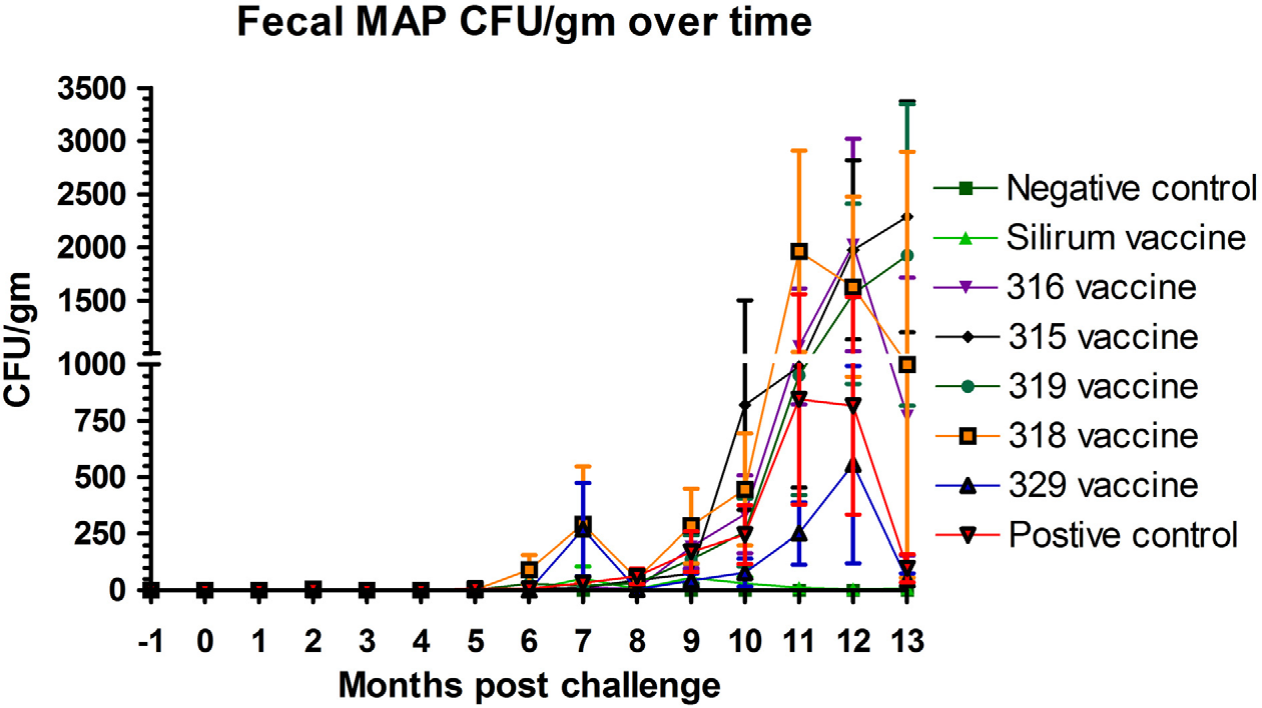
**Monthly fecal culture results on Herrold's Egg Yolk medium with and without mycobactin J throughout the study.** No positive samples were detected in the non-challenged control group. (Error bars represent standard error of the mean, −1 is baseline bleeding, 0 is challenge date and 1–13 represents months post-challenge).

The effect of vaccination on fecal shedding was determined by both fecal culture and fecal PCR. There was an initial spike of fecal shedding (Figure 3) at 7 months post-challenge that subsided by 8 months post-challenge, but returned by 9 months and persisted to the end of the study in most groups with the exception of group 2 (Silirum® vaccine). The Silirum® vaccine (group 2—Figure 3) showed a reduction in fecal CFU/g at all time points post-challenge as compared to the positive control group (8) although this reduction was not statistically significant (*P* = 0.06). The 315, 316, 318, and 329 vaccines (groups 3–6) had similar levels of fecal shedding as compared to the positive control group and the 329 vaccine (group 7) had slightly less shedding (but comparable; *P* = 0.10) than the positive control group.

**Figure 4:**
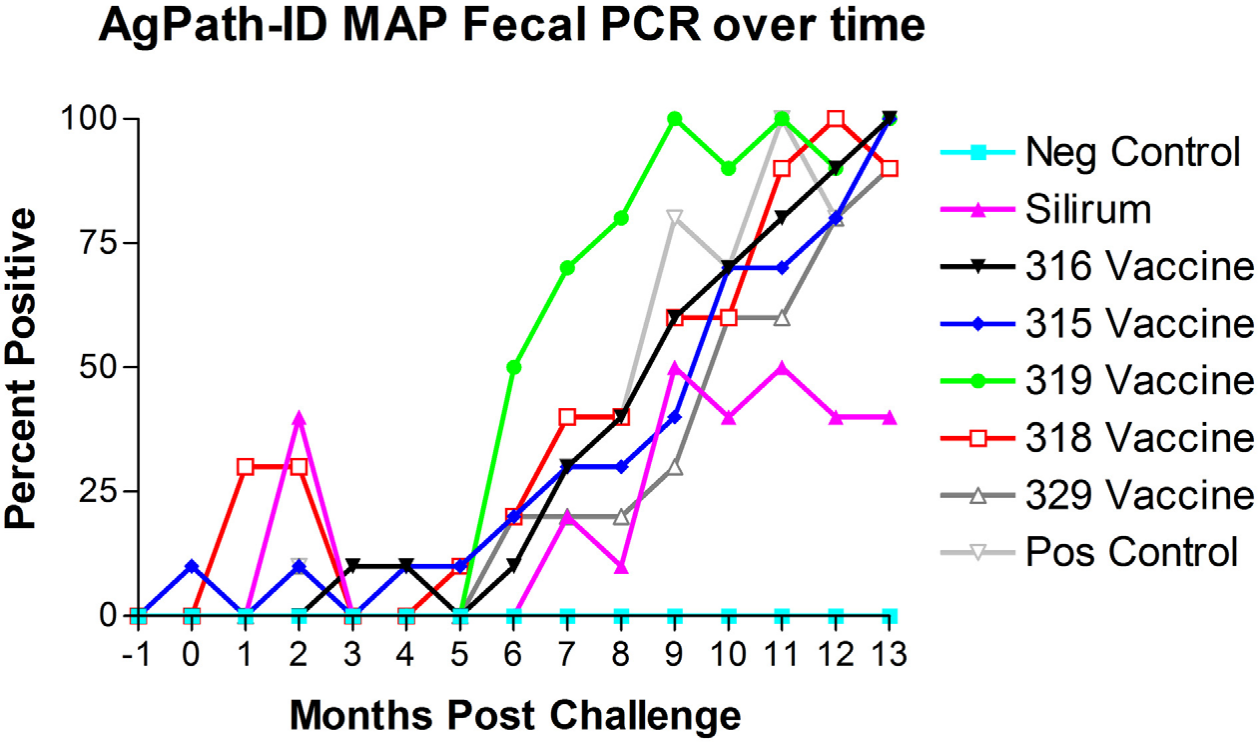
**Monthly fecal AgPath-ID™ PCR results throughout the study.** No positive samples were detected in the non-challenged control group. (−1 is baseline bleeding, 0 is challenge date and 1–13 represents months post-challenge).

AgPath-ID™ MAP PCR was performed on the monthly fecal specimens obtained from all kids in the study. Both fecal PCR CT values and Positive/Negative results were obtained. Figure 4 shows the results of fecal PCR over time represented as percent positive samples. No PCR positive samples were detected in the negative control group (group 1) throughout the study. Sporadic PCR positive samples were detected from 0 to 5 months post-challenge in several treatment groups. Beginning at 5–6 months post-challenge all challenged groups progressively increased in PCR positivity. At 9 months post-challenge the Silirum® vaccinated group percent positivity leveled off at 40–50% and remained near this level till the end of the study, while all other challenged groups continued to increase to near 100% positivity by the end of the study.

To determine the persistence of the vaccine strains on fecal shedding, primers were designed to amplify target regions specific to each knockout mutant vaccine yet able to distinguish vaccine from challenge strains based on product size. PCR amplifications were performed on selected colonies obtained from fecal culture of multiple goats from each group given one of the live mutant vaccines. Only the challenge strain was identified from the amplified products. The vaccine strains were not among any of the colonies suggesting that they were not persistent in the goats. For *in vitro* cultures, amplification was not successful using primers from vaccines 318 and 329 due to the instability of the corresponding mutations (Raul G. Barletta and Adel M. Talaat, unpublished results). Amplicons specific for insertions in other vaccine candidates were identified correctly.

### Effects on tissue colonization of MAP

In intestinal tissues, kids vaccinated with Silirum® (group 2) had dramatically lower mean numbers of MAP CFU/g than the positive control group (8) (*P*-value range 0.0013–0.1044). As shown in Table 4, mean MAP CFU/g cultured from each tissue individually were reduced by 10+ fold in most tissues from Silirum® vaccinated kids. Kids vaccinated with the 315 and 319 vaccines (groups 4 and 5) had in general higher (although not statistically significant) CFU/g in most tissues than those of the positive control group (group 8). In general, either no or very low MAP CFU/g were cultured from non-intestinal tissues (liver, spleen, tonsil, and submandibular lymph nodes) in all challenged groups. The highest numbers of CFU/g were cultured from intestinal tissues including mesenteric and ileocecal lymph nodes, ileum, jejunum and duodenum, but much lower numbers were cultured from spiral colon.

**Table 4:** **HEYM tissue culture (CFU/gm) means and ranges, by vaccine group (rounded to nearest whole number)**.

**Tissues**	**(−) control**	**Silirum®**	**316 vaccine**	**315 vaccine**	**319 vaccine**	**318 vaccine**	**329 vaccine**	**(+) control**
**Group**	**1**	**2**	**3**	**4**	**5**	**6**	**7**	**8**
Liver	0	0	0	37	13	1	76	1
	(0–0)	(0–0)	(0–2)	(0–197)	(0–101)	(0–3)	(0–749)	(0–8)
Spleen	0	0	1	5	1	0	0	1
	(0–0)	(0–0)	(0–6)	(0–43)	(0–5)	(0–2)	(0–0)	(0–13)
Tonsil	0	0	1	1	0	0	0	0
	(0–0)	(0–0)	(0–3)	(0–6)	(0–2)	(0–0)	(0–2)	(0–0)
Submandibular LN	0	0	0	14	0	0	0	0
	(0–0)	(0–0)	(0–0)	(0–102)	(0–2)	(0–2)	(0–0)	(0–0)
Mesenteric LN-1	0	55	511	1095	964	651	480	772
	(0–0)	(0–477)	(0–1440)	(2–1440)	(59–1440)	(0–1440)	(0–1440)	(2–1440)
Mesenteric LN-2	0	86	616	797	926	620	653	828
	(0–0)	(0–757)	(0–1440)	(0–1440)	(30–1440)	(0–1440)	(0–1440)	(11–1440)
Ileocecal LN	0	63	412	770	980	727	536	589
	(0–0)	(0–259)	(0–1440)	(0–1440)	(13–1440)	(0–1440)	(0–1440)	(16–1440)
Ileum	0	5	240	585	564	397	175	362
	(0–0)	(0–22)	(0–1440)	(0–1440)	(0–1440)	(0–1440)	(0–1440)	(0–1440)
Jejunum-1	0	33	448	893	958	569	323	870
	(0–0)	(0–98)	(0–1440)	(2–1440)	(130–1440)	(0–1440)	(0–1440)	(19–1440)
Jejunum-2	0	87	614	1053	858	583	351	720
	(0–0)	(0–786)	(0–1440)	(0–1440)	(5–1440)	(0–1440)	(0–1440)	(53–1440)
Jejunum-3	0	7	474	883	598	804	454	619
	(0–0)	(0–19)	(0–1440)	(0–1440)	(18–1440)	(0–1440)	(0–1440)	(10–1440)
Duodenum	0	1	107	722	422	192	91	353
	(0–0)	(0–5)	(0–885)	(0–1440)	(0–1440)	(0–1440)	(0–878)	(0–1440)
Spiral colon	0	0	44	227	204	27	4	9
	(0–0)	(0–0)	(0–235)	(0–1440)	(0–1440)	(0–168)	(0–22)	(0–78)

LN, Lymph node.

AgPath-ID™ MAP PCR was performed on the tissue specimens obtained from all goats at necropsy. Both tissue PCR CT values and Positive/Negative results were obtained. Table 5 shows the tissue PCR results represented as percent positive samples. No PCR positive samples were detected in the negative control group (group 1) throughout the study. Only the Silirum® vaccinated group (group 2; *P* < 0.05) and 329 vaccinated group (group 7; *P* < 0.05) showed reductions in percent positivity as compared to the positive control group (group 8).

**Table 5:** **Tissue AgPath-ID MAP PCR percent positive**.

**Tissues**	**(−) control**	**Silirum®**	**316 vaccine**	**315 vaccine**	**319 vaccine**	**318 vaccine**	**329 vaccine**	**(+) control**
**Group**	**1**	**2**	**3**	**4**	**5**	**6**	**7**	**8**
Liver	0	0	90	100	60	60	30	70
Spleen	0	0	70	90	40	50	40	60
Tonsil	0	0	70	70	80	50	40	90
Submandibular LN	0	10	90	60	60	50	30	60
Mesenteric LN-1	0	70	100	70	100	100	90	100
Mesenteric LN-2	0	70	100	70	100	90	90	90
Ileocecal LN	0	60	90	80	100	90	50	100
Ileum	0	50	90	100	90	90	80	100
Jejunum-1	0	60	100	100	90	80	80	100
Jejunum-2	0	70	100	100	100	100	90	100
Jejunum-3	0	70	100	90	100	100	90	100
Duodenum	0	0	100	100	80	50	50	70
Spiral Colon	0	0	80	100	60	60	50	90

LN, Lymph node.

### AGID

**Figure 5:**
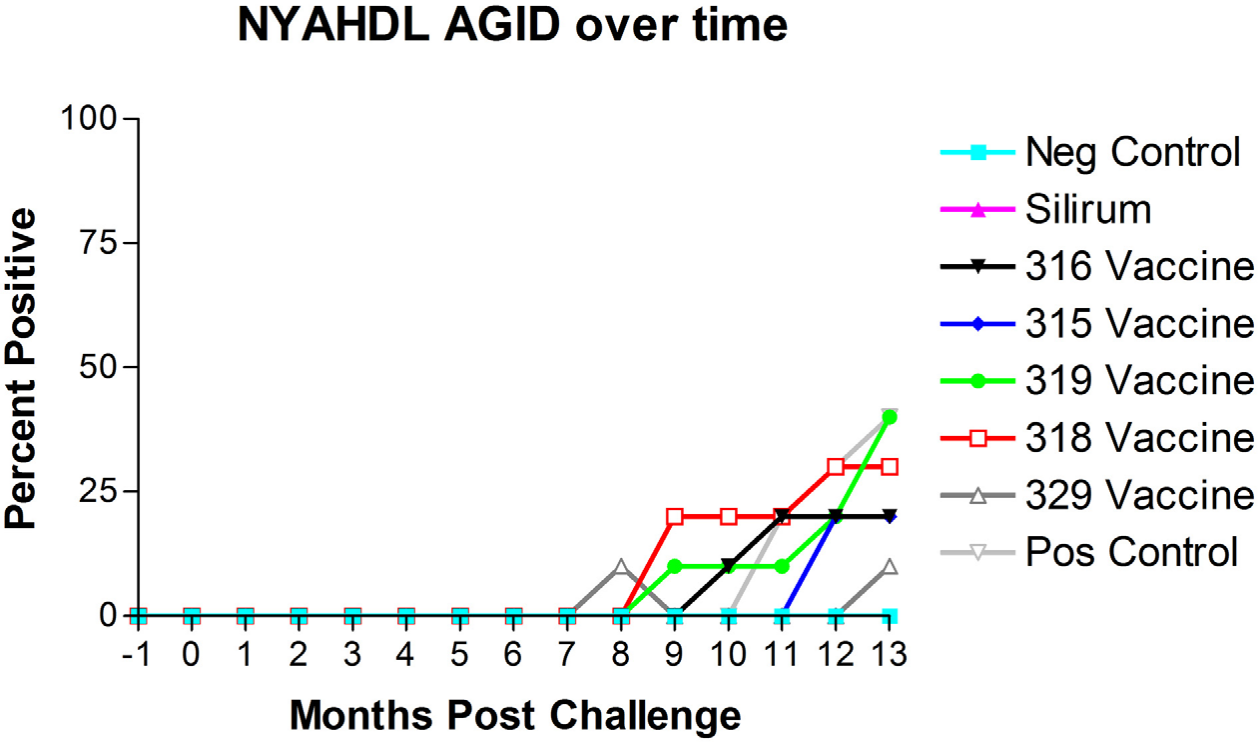
**New York Animal Health Diagnostic Laboratory (NYAHDL) AGID test results over time.** The NYAHDL AGID is an agar gel immunodiffusion test for measuring serum antibody to *Mycobacterium avium* subsp. *paratuberculosis.* Note that no animals in the Silirum® vaccine group and only 1 animal in the 329 vaccine group (group 7) became AGID positive. (−1 is baseline bleeding, 0 is challenge date and 1–13 represents months post-challenge).

The results of AGID testing are shown in Figure 5. No non-challenged kids (group 1) became AGID positive during the study. All kids in challenged groups remained negative on the AGID test until 8 months post-challenge. The percentage of AGID positive animals in remaining challenged groups increased from 8 months to 13 months. However, no animals in group 2 (Silirum® vaccine) had become positive by the end of the study, and only 1 kid in group 7 (329 vaccine) became positive on the AGID test at 13 months post-challenge.

### ELISA

**Figure 6:**
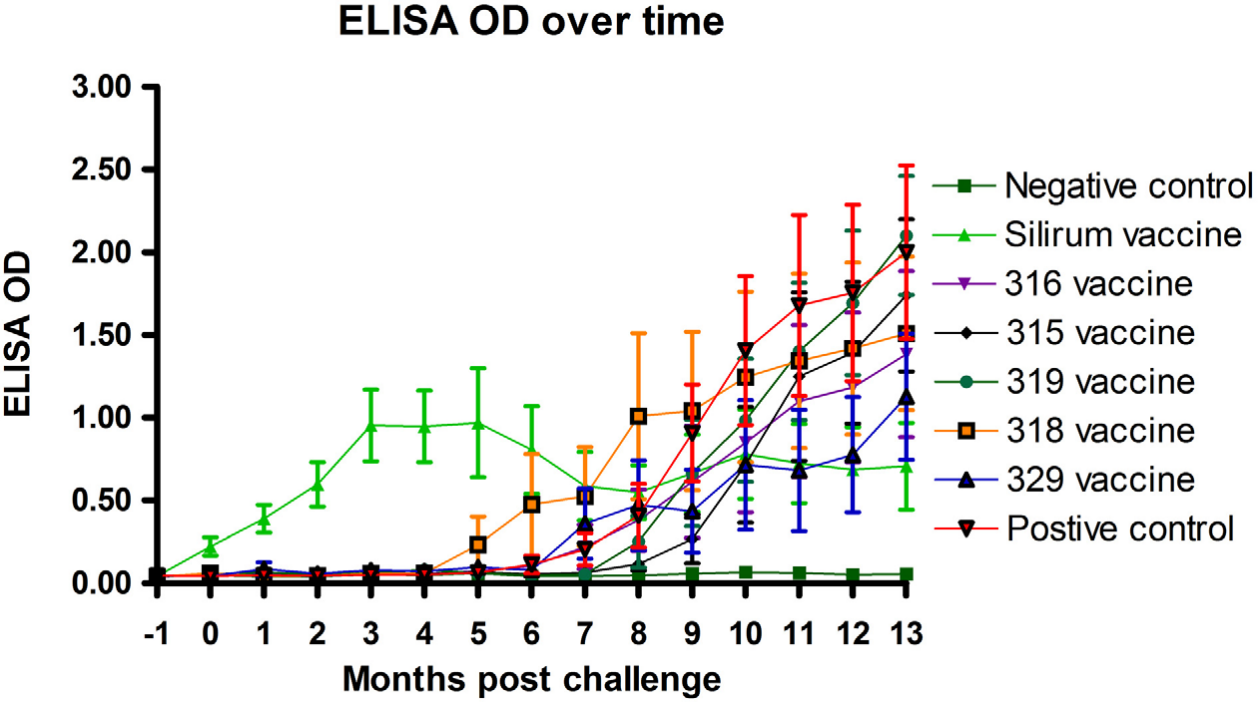
**Parachek® ELISA optical density (OD) readings over time.** Note the almost immediate increase in ELISA OD for the Silirum® vaccinated group, the progressive increase in ELISA OD in most vaccine groups beginning around 5 months post-challenge and the leveling off of the Silirum® vaccinated group OD values at 9–13 months post-challenge. (Error bars represent standard error of the mean, −1 is baseline bleeding, 0 is challenge date and 1–13 represents months post-challenge).

Optical density measurements of the Parachek® ELISA over time are shown in Figure 6. Sensitivity values of the ELISA test, from 1 to 7 months, 1 to 9 months, and 1 to 15 months, for each of the six vaccines, are shown in Table 6; the cutoff value was set at ELISA_OD = 0.25. If the ELISA test was positive at any time point in the interval, the animal was considered to be positive. Sensitivity improved as the study progressed. Vaccination with the Silirum® vaccine (group 2) resulted in an almost immediate rise in ELISA OD values that leveled off at 3–4 months post-challenge, tended to fluctuate slightly and trend slightly lower, but persisted throughout the study. None of the sham-vaccinated non-challenged kids (group 1) developed significant OD values. ELISA OD values in the remaining groups (groups 3–8) began to rise between 4 and 6 months and continued to rise through the remainder of the study as the disease progressed. Sensitivity and specificity are usually used to assess test performance. In this study, the same test (ELISA) was used in different vaccine groups to see how it performed with different vaccines (Table 6). That is, the test *per se* is constant, but the vaccines (experimental treatments) are variable.

**Table 6:** **Sensitivity (%) of the Parachek® ELISA, by vaccine. The cutoff value was set at ELISA_OD = 0.25**.

**Vaccine (group)**	**Months 1–7**	**Months 1–9**	**Months 1–15**
Silirum® (2)	100	100	100
316 (3)	0	20	60
315 (4)	0	0	60
319 (5)	10	10	100
318 (6)	10	30	50
329 (7)	10	30	50

### PPD skin tests

**Figure 7:**
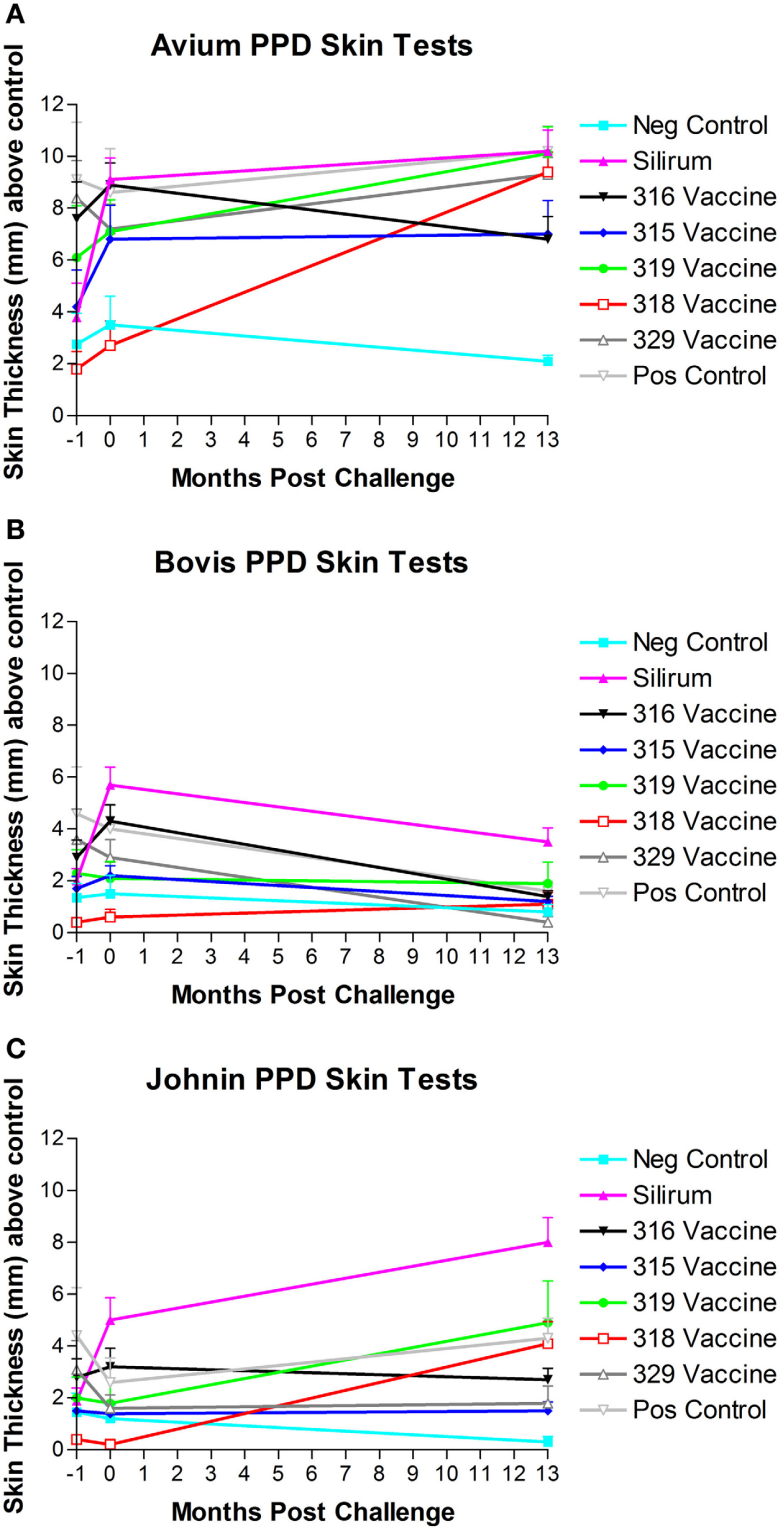
**(A–C)** PPD skin test results over time. Note the strong skin responses to *M. avium* **(A)** and Johnin **(C)** PPD in multiple groups, and the generally lower responses to *M. bovis* **(B)** PPD with the exception of the Silirum® and 316 vaccines. (Error bars represent standard error of the mean, −1 is baseline bleeding, 0 is challenge date and 1–13 represents months post-challenge).

Three comparative cervical PPD skin tests including *M. avium* PPD, *M. bovis* PPD, and Johnin PPD were performed at the beginning of the study, after vaccination but prior to challenge and before necropsy (13 months post-challenge). Results of PPD skin testing over time are shown in Figures 7A–C. When a 2.0 mm cutoff above normal skin thickness was used for positive skin test reactions, spontaneous false-positive PPD skin test reactions were common in all groups for *M. avium*. Vaccination resulted in false-positive PPD skin test reactions for *M. avium* PPD in the Silirum®, 315, 316, and 319 vaccinated groups, and Johnin PPD in the Silirum® and 316 vaccinated groups. When a 2.0 mm cutoff above normal skin thickness was used for positive skin test reactions, false-positive reactions for *M. bovis* were detected particularly in the Silirum® vaccinated group and to a lesser extent in the 316 and 329 groups, and remained consistently negative in the three time points evaluated for vaccines 315 and 318.

Overall, there was a significant difference in ranking of treatment groups in the skin tests (combined). Goats in the 318 and 315 groups had lower skin thickness (across all tests) than did goats in the positive control group (*P* = 0.0011 and *P* = 0.0037, respectively).

## Discussion

Development of an effective control strategy against Johne's disease in dairy herds remains an elusive goal despite the presence of commercial vaccines for cattle (Mycopar®) and small ruminants (Silirum®). With the development of molecular and genomic tools to study MAP, the causative agent of JD, we sought to take a rational approach for developing live attenuated vaccine by focusing on MAP mutants that were shown to be attenuated during infection (Shin et al., 2006). Drs. Barletta and Talaat have now generated stable deletion mutants that could be tested in a second trial with the methods reported in this manuscript. However, at the time this phase of the efficacy project was started genetically stable versions of these mutants were not yet available. The primary goals of this study were to (1) to evaluate the efficacy of 5 attenuated strains of MAP as vaccine candidates compared to a commercial control, and (2) to validate the AMSC Johne's disease goat challenge model (see Hines et al., 2007b). However, another goal of our study was to develop an easy-to-administer oral vaccine against JD in comparison to the commercially available vaccines administered via subcutaneous injection.

All challenged baby goats (kids) in the study had lesions compatible with JD suggesting none of the vaccines prevented infection and that the goat model used in this study is highly efficient (100%) in producing active JD in challenged goats. As expected, none of the unchallenged kids had gross or microscopic lesions compatible with JD. The gross and microscopic lesions observed in this study were generally similar to those described previously (Clarke, 1997; Storset et al., 2001; Munjal et al., 2005; Hines et al., 2007a).

A key disadvantage of currently licensed vaccines is the development of skin lesions at the site of inoculation. In this study, ante-mortem vaccine site reaction was not an issue with the experimental vaccines as they were given by the oral route. However, the control vaccine used in our study produced significant tissue reaction at the injection site that persisted to the end of the study in approximately 40% of the kids in group 2. The control vaccine group (group 2, Silirum®) showed a marked reduction in fecal CFU/g at all time points post-challenge and a lesser reduction in fecal CFU/g was found with one of the experimental vaccines (vaccine 329). However, wide variation in fecal and tissue MAP CFU/g occurred both among and within groups, which reduced statistical power and probably indicates the need for evaluation of larger numbers of animals in future studies. A similar pattern was observed for the lesions scores and tissue colonization data obtained following histological and bacteriological analyses. It is noteworthy to mention here that the mean MAP CFU/g cultured from individual tissues were reduced by 10+ fold in most tissues from control vaccinated kids (group 2, Silirum®). These data also suggest that spread of MAP infection is limited beyond the intestine in the Silirum® vaccinated goats. Our findings are consistent with prior studies (Saxegaard and Fodstad, 1985; Fridriksdottir et al., 2000; Reddacliff et al., 2006; Hines et al., 2007a) that show that “whole cell” bacterins can reduce fecal shedding of MAP. However, this relationship was not evident in other studies (Wentink et al., 1994; Koets et al., 2000, 2006). It is possible that subcutaneous inoculation of the experimental mutant vaccines could provide a similar shedding pattern to that observed in the control vaccine group.

Real time PCR using the AgPath-ID™ PCR test (Figure 4) was able to detect the presence of MAP within feces significantly earlier than HEYM fecal culture and likely detected the 315 vaccine strain at 0 months post-challenge (prior to administration of the challenge). MAP was detected by PCR in some animals from the Silirum®, 315 and 318 vaccinated groups at 2–3 months post-challenge that is likely due to the challenge MAP strain, but in the 315 and 318 vaccinated groups the vaccine strains MAP could have still been present and contributed.

The dependence of JD control programs on diagnostic tests based on the immune responses generated after exposure/infection with MAP complicates the widespread use of JD vaccines in the field. All kids in challenged groups remained negative on the AGID test until 8 months post-challenge, none of the control vaccinated kids ever became positive on AGID and only one animal in the 329 vaccine group became positive on AGID. These findings demonstrate the limited usefulness of the AGID test as it tends to only detect the more advanced cases of JD in goats. Subcutaneous vaccination with the Silirum® vaccine (group 2, positive control vaccine) resulted in an almost immediate rise in ELISA OD values after vaccination that leveled off at 3–4 months post-challenge, tended to fluctuate slightly and trend slightly lower over time, but persisted throughout the study. None of the sham-vaccinated non-challenged kids (group 1) developed significant OD values. ELISA OD values in the remaining groups (groups 3–8, experimental vaccines and positive control group) began to rise between 5 and 8 months post-challenge and continued to rise through the remainder of the study as the disease progressed. Since all experimental mutant vaccines were administered by the oral route, this may explain why ELISA OD values in these vaccines were initially subdued and humoral immunity was delayed until 5–8 months post-challenge.

Another complication of using whole bacterin or live attenuated vaccine is the cross reactivity to skin testing for *M. bovis* infection or for even natural infection with field isolates of MAP. Our analysis indicated that spontaneous false-positive PPD skin test reactions were common in all groups for *M. avium* even prior to vaccination. This is not uncommon due to the high exposure to environmental mycobacteria within the southeastern US which complicates the interpretation of skin test and γ-interferon results. Vaccination resulted in false-positive PPD skin test reactions for *M. avium* PPD in control, Silirum®, 315, 316 and 319 vaccinated groups, and Johnin PPD in the Silirum® and 316 vaccinated groups. When a 2.0 mm cutoff above normal skin thickness was used for positive skin test reactions, false-positive reactions for *M. bovis* were detected particularly in the Silirum® vaccinated group and to a lesser extent in the 316 and 329 groups. These findings indicate that several of the vaccines and particularly the Silirum® vaccine significantly impact the results of PPD skin testing and produce false positive results with the tuberculosis (*M. bovis*) skin test that may impact tuberculosis control testing programs.

Other reports evaluating the performance of attenuated mutant MAP strains that include evaluation of live attenuated vaccines in a ruminant model are sparse within the literature. A recent report evaluated a MAP *leuD* mutant as a vaccine candidate in a goat challenge model (Faisal et al., 2013). However, the study by Faisal and coauthors did not utilize the suggested standardized challenge parameters proposed for goat challenge models by the AMSC. Their study did not include a negative non-challenged control group to prove that none of the animals were infected before or during the study, Mycopar® was used instead of Silirum® as a positive control vaccine, a non-K10-like MAP strain was used as the challenge MAP strain, and persistence of the attenuated vaccine strain was not evaluated. In our hands, not all of our vaccine constructs were stable during culturing, most likely because of their nature as transposon mutants. In future experiments, using a stable mutant constructed by allelic exchange, for example, could provide a better alternative. Therefore, direct comparison of results between these two studies is not possible due to the number of extraneous variables involved.

## Conclusions

We found that (1) The AMSC proposed standard goat MAP challenge model is highly efficient in producing infection in goats and is a valid model for JD challenge and/or vaccine efficacy studies; (2) none of the control or experimental oral vaccines in this study prevented MAP infection, although administration of the 329 vaccine lowered the incidence of infection and reduced lesion scores; (3) only the control vaccine (Silirum®) showed a clear reduction in lesion score, MAP fecal shedding and tissue colonization, but it induced tissue damage in the site of inoculation that persisted in 40% of the animals; (4) based on evaluation of lesion scores, fecal shedding and tissue colonization the relative performance ranking of the vaccines evaluated in this study Silirum® was the best performer, then the 329 vaccine, 318 vaccine, 316 vaccine, 315 vaccine and the 319 vaccine was the worst performer; and (5) oral delivery may not be a viable alternative to deliver live attenuated vaccines against JD. Our results suggest that although vaccination with Silirum® does not prevent infection or eliminate MAP fecal shedding, it reduces presence of JD gross and microscopic lesions, slows progression of disease, reduces fecal shedding and reduces tissue colonization.

## Author contributions

Murray E. Hines II was the principle investigator in this study who was mostly responsible for the conception, design and execution of this study. He was involved in all aspects of the project and performed most of the writing of grants and manuscripts. Sue E. Turnquist was involved in the overall project design, execution of the project, acquisition of samples/data and interpretation of necropsy data. Marcia R. S. Ilha was involved in the overall project design, execution of the project, acquisition of samples/data and evaluation of necropsy data. Sreekumari Rajeev was involved in the overall project design, execution of the project and acquisition of samples/data. Arthur L. Jones was involved with the overall project design, sample/data acquisition and assisted the project with veterinary support. Lisa Whittington was the primary person in the project responsible for sample and data acquisition. Raúl G. Barletta contributed several vaccine strains (315, 316, 318, and 319), developed the PCR detection method for the strains provided and contributed to the overall trial design and participated in manuscript writing. John P. Bannantine was involved with the overall project design and performed the PCR analysis to determine the survival of the mutant vaccine strains in this project. Yrjö T. Gröhn was the statistician for the project and was involved with the overall project design, data analysis/interpretation and manuscript writing for the project. Robab Katani and Lingling Li were involved with production and blinding of the mutant vaccine strains. Adel M. Talaat was involved in the overall project design, provided vaccine strain 329 and contributed to the manuscript writing. Vivek Kapur is the principle investigator of JDIP and was involved with the overall design of the project, blinding of the mutant vaccine strains and interpretation of the data.

### Conflict of interest statement

Pfizer Animal Health (now Zoetis) provided the Silirum® vaccine used on the vaccine control group in this study free of charge and made a donation of $10,000 to support this study. Adel M. Talaat declares his affiliation with Pan Genome Systems, Inc. Neither company was allowed to contribute to or influence the design, conduction, interpretation or evaluation of this study and the data/results from this study have not been provided to them. No patents or copyrights are involved.
